# Complementary and alternative medicine use among adults in Enugu, Nigeria

**DOI:** 10.1186/1472-6882-11-19

**Published:** 2011-03-04

**Authors:** Jane-lovena E Onyiapat, Ijeoma L Okoronkwo, Ngozi P Ogbonnaya

**Affiliations:** 1 Department of Nursing Sciences, College of Medicine, University of Nigeria, Enugu Campus, Nigeria

## Abstract

**Background:**

Attention and interest in the use of Complementary and Alternative Medicine (CAM) has been reawakened globally. Evidence from studies carried out in different parts of the world has established that CAM use is very common and varies among populations. This study investigated the use of CAM among adults in Enugu urban, irrespective of their health status. It provided information on the prevalence of CAM use, forms of CAM remedies used and reasons for utilizing them

**Methods:**

The study areas were three local government areas in Enugu urban of Enugu State. Cross-sectional survey using questionnaires were administered to randomly selected households. All consenting participants were used for the study

**Results:**

732 participants (37.2% males and 62.8% females) were used for the study. Ages ranged from 18 - 65 years. 620 (84.7%) of the adult population have used CAM ranging from one single type to twenty different types while 112 (15.3%) have not used any form of CAM. The most commonly used CAM product was the biological products, followed by prayer/faith healing. Major reasons for using CAM include their natural state and also for health promotion and maintenance.

**Conclusion:**

There is need for adequate policy formulation and regulation to ensure safety and efficacy of CAM products. Measures to ensure rational use of CAM should be instituted.

## Background

It has been observed that many adults use different health products or measures under the umbrella of CAM. Reason for use could either be that they are imported or approved by National Agency for Food, Drug Administration and Control (NAFDAC), or they are believed to promote health, with little or no knowledge about the compositions, uses and side effects. NAFDAC is a parastatal of the Federal Ministry of Health established to regulate and control the manufacture, importation, exportation, distribution, advertisement, sale and use of food, drugs, cosmetics, chemicals, medical devices and packaged water.

The National Centre for Complementary and Alternative Medicine (NCCAM) [1] defines CAM as "a group of various medical and health care systems, practices, and products that are not presently considered to be an aspect of conventional medicine". Complementary interventions are healthcare approaches used in conjunction with conventional interventions, whereas alternative medicines are used in place of conventional medicine [2]. CAM as used in this study is an umbrella term which includes traditional medicine in-addition to other western remedies and/or strategies that are not conventional e.g. forever living products, Tianshi etc

CAM is a growing area of health care within developed and developing countries and is increasingly popular with consumers and professionals [3]. In the past decade, attention and interest in the use of CAM has been reawakened globally. Evidence from studies carried out in different parts of the world has established that CAM use is very common and varies among populations [4,5] and the nature of what is consumed under the umbrella of CAM varies in form and number in different parts of the world. The most popular include herbs, acupuncture, non vitamin, non mineral, natural products, faith/prayer healing, among others [2,6,7].

The global situation as reported in the WHO tradition medicine strategy 2002 - 2005 showed a CAM use prevalence rate of Belgium (31%), China (40%), Colombia (40%), USA (42%), Australia (48%), France 49%, India (65%), Canada (70%) and Chile (71%) [5]. The same report observed that in Africa, up to 75% of people living with HIV/AIDS use traditional medicine. A literature review on the use of CAM for cancer patients in a number of European countries suggest that CAM is popular among cancer patients with 35.9% using some form of CAM (range among countries 14.8% to 73.1%) [8].

In developing countries, the prevalence of CAM use has been described by very few studies. Available literature indicates that few studies have evaluated the prevalence of CAM use in the general population [7]. A prevalence rate of 38.5% was recorded among the general population of Indians living in Chatsworth, South Africa with the most common being herbs and spiritual healing [9]. A study on self reported use of CAM in Jeddah western Saudi observed that over 80% of the populations in developing countries depend on CAM products and/or traditional healing modalities, including herbal remedies, for health maintenance and therapeutic management of disease [10]. However, other studies on CAM use in developing countries were among women with breast cancer in Singapore where out of 146 patients analysed, 58.6% consumed CAM along with chemotherapy with 72.1% consuming CAM even before diagnosis was made [11].

In Nigeria, studies carried out on prevalence of CAM use were among cancer patients. Out of the 160 patients interviewed on the use of CAM, a prevalence rate of 65.0% was recorded among cancer patients at the University of Nigeria Teaching Hospital (UNTH) Enugu, Nigeria [7] and among children with chronic health conditions [12].

The various forms of CAM used were herbs (51.9%), faith/prayer healing (49.4%), aloe vera (23.1%), forever living products (16.3%), medicinal tea (14.4%) and black stone (12.5%). It was also reported that while more women were non-CAM users, the use of CAM was not affected by age, marital status, level of education, religious affiliation and socioeconomic status.

Demographic relationships with the prevalence of CAM use have also been cited [2,7,9]. In developed countries, younger adults, female gender, higher level of education, higher income and social class seem to be associated with more frequent use of CAM. Barnes et al reported that CAM use was more prevalent among women, adults aged 30 - 69 years, higher level of education, higher income and people with one or more health conditions [2]. In contrast, studies carried out in developing countries found no significant relationships between demographic profile such as age, gender, marital status and use of CAM [7,9]. They however, documented an inverse relationship between use of CAM and socioeconomic status.

Many reasons have been attributed to the use of CAM which include to treat or prevent diseases and to improve the quality of life [5]. Those who utilize it have long standing conditions for which conventional medicine cannot provide effective relief, either due to ineffectiveness of therapy or because it causes adverse effects. A study on herbal use among US elderly national health interview survey, revealed that many use herbal products because it is perceived to be natural, with fewer side effects [13,14]. The availability of these products in the supermarkets, health food shops and internet has increased the perception that they are safe. Reports also indicate that people who use CAM measures are looking for ways to improve their health and wellbeing [15]; or to relieve symptoms associated with chronic, even terminal illnesses or the adverse effects of conventional medicine[16,17]. Other reasons include having a holistic health approach and having greater control over one's own health [18].

In Nigeria, although the use of traditional herbs and remedies are well known and relatively common, the use of CAM in the general adult population irrespective of their health status is unknown. This present study is therefore a preliminary to providing baseline data on prevalence of CAM use among adult population of Nigerians and will identify forms of CAM common to Nigerians.

## Methods

### Study area

The study area was drawn from three local government areas in Enugu urban. Enugu urban is the capital of Enugu State which is made up of Enugu North, South and East Local Government Areas (L.G.As). Being the capital city it has a large concentration of adults who are engaged in various activities.

### Population

The population of the study comprised of adults between the ages of 18 to 65, who were residing in Enugu urban at the time of study. As at 2006, Enugu urban had a population of 722,644 [19]. The upper age limit of 65 years was used because the age limit for work force retirement falls within 60-65 years depending on the work sector. This age group 18-65 years were therefore assumed to be capable of taking decisions and actions concerning their health.

### Sample

A sample size of 1000 was determined using Yaro Yamene formular. This is a statistical technique used to determine sample size for a known population [20]. To select the respondents, a multistage sampling technique was used. The first stage involved clustering Enugu urban into three L.G.As. In stage two, two residential areas were selected from each of the L.G.A using simple random sampling, giving a total of six residential areas: Trans-Ekulu, Emene, Uwani, Maryland, New Heaven and Coal Camp. The third stage involved the use of simple random sampling to select 30 out of the estimated 259 streets in the residential areas. Systematic random sampling was used in the fourth stage to select 1000 houses from the selected streets. Thus, one adult from each house constituted the sample.

### Data collection

The only instrument for data collection was a structured questionnaire developed by the researchers which was administered to each participant. Research assistants were trained to facilitate administration. For participants who were not literate, the questionnaire was interpreted to them. The questionnaire was pretested amongst 20 adult residents of a peri-urban community near Enugu who were not involved in the study. Results were used to improve the language used in the questionnaire and mode of questioning. The questionnaire contained questions on socio-demographic profile of respondents, their health status, and also examined prevalence of CAM use, forms and reasons for use.

The protocol was approved by the Enugu State Ministry of Health ethical committee and informed consent was obtained from all the participants who were willing to take part in the study (Additional file 1).

### Data analysis

Data generated were analyzed using frequencies and percentages while chi square was used to determine association between socio demographic and economic characteristics and CAM use with the level of significance at p = 0.05.

## Results

The number of properly filled questionnaires available for data analysis was 732 out of the 1000 copies distributed giving a return rate of 73.2%. Some copies of the completed questionnaires were mutilated, while others were wrongly filled and were discarded as they could not be used for data analysis. Thus 732 respondents constituted the sample size. Out of this number, 272 (37.2%) were males and 460 (62.8%) females. 84.7% were CAM users while 15.3% were non CAM users. Mean age of participants was 37.6 (SD = 1.28)

Table 1 compares the demographic and economic characteristics of CAM users and non users. More males used CAM than their female counterparts (p = 0.004). Marital status was found to be significant with usage of CAM (p = 0.005) with more of the 'married' group being CAM users. Level of income was found to be statistically significant with CAM usage indicating an increase with low income status. Although a greater number of the age groups between 26-41 years used more CAM than other age groups, these differences were not statistically significant. Participants who had no formal education were more likely to have used CAM than those who had higher education. Again, these differences were not statistically significant.

**Table 1:** Demographic characteristic of CAM users and non users

Parameter	CAM Users	Non CAM Users	Significance*
**SEX**			
**Male**	244(89.7%)	28(10.3%)	0.004*
Female			
**Mean age**	**37.4**	**38.7**	**0.267**
**Marital status**			0.005*
Never married	213(34.4%)	33(29.5%)	
Married	370(59.7%	60(53.6%)	
Divorced/separated	9(1.4%)	3(2.7%)	
Widow/widower	28(4.5%)	12(10.7%)	
No entry	0	4(3.5%)	
**Level of**			**0.056**
**education**			
No formal	4(0.7%)	1 (0.9%)	
Primary	25(4.0%)	7 (6.3%)	
Post primary	226(36.5%)	46 (41.1%)	
Tertiary	363(58.5%)	54 (48.2%)	
No response	2(0.3%)	4 (3.5%)	
**Level of income**			0.000*
Low income(N50,000/month	479 (77.2%)	63 (56.2%)	
Middle income(N50,000-100,000/month	117 (18.9%)	30 (26.8%)	
High income(N100,000 and above	24(3.9%)	6 (5.4%)	
No response	0	13 (11.6%)	
**Religion**			
Christianity	616(99.3%)	111 (99.1%)	
Moslem	1(0.2%)	1 (0.9%?)	
Traditional religion	3(0.5%)	0	

Considering the overall health status of the users as shown in Table 2, (33.9%) of CAM users assessed their health status as satisfactory as opposed to 34.8% of non CAM users. On the other hand, (11.9%) of CAM users classified their health status as poor as against (3.6%) of non CAM users (Table 3).

**Table 2:** Health Status of Respondents

Options	Users (n = 620)	Non-Users (n = 112)
Satisfactory	210 (33.9%)	39 (34.8%)
Good	309 (49.8%)	62 (55.4%)
Poor	74 (11.9%)	4 (3.6%)
Seriously sick	27 (4.4%)	7 (6.2%)

**Total**	**620 (100.0%)**	**112 (100.0%)**

**Table 3:** Number of CAM remedies used n = 620

Number of CAM	Frequency	Percentage
1 - 5	440	71.0%
6 - 10	107	17.2%
11 - 15	71	11.5%
16 - 20	2	0.3%

**Total**	**620**	**100.0%**

Most of the respondents (71.0%) used one to five different forms of CAM, while only (0.3%) used between sixteen to twenty forms.

The most commonly used form of CAM (Table 4) was the biological - based treatments (56.0%) with honey being the most frequently used product in this category. Other forms used include Spiritual therapy (49.4%) with faith/prayer healing being the most frequently used, physical therapy (22.1) with massage as the most frequently used, others (33.9%) with black stone as the most frequently mentioned (see Table 5 for the breakdown of the categories).

**Table 4:** Forms of CAM remedies used by respondents *

Forms of CAM	Frequency	Percentage
Biological products	347	56.0%
Spiritual therapy	306	49.4%
Physical therapy	137	22.1%
Alternative medicine	45	7.3%
Others	140	26.6%

*Multiple responses given

**Table 5:** Breakdown of CAM products used by respondents *

Types	Frequency of use & %	Types	Frequency of use & %
**Biological Products;**		**Alternative Systems:**	
Herbal drugs	335 (54.0%)	Chinese medicine	45 (7.3%)
Forever living product	148 (23.9%)	Acupuncture	20 (3.2%)
Tuja 1000	37 (6.0%)	Homeopathy	27 (4.4%)
Aloe vera	175 (28.2%)	**Physical Therapy/body Manipulation**	
GNLD product	120 (19.4%)	Osteopathy	29 (4.7%)
Tianshi products	101 (16.3%)	Massage	137 (22.1%)
Formor products	16 (2.6%)	Manual healing	33 (5.3%)
Noni juice	21 (3.4%)	**Others**	
Medical Tea	45 (7.3%)	Local Surgery	31 (5.0%)
Green Tea	36 (5.8%)	Ritual sacrifice	11 (1.8%)
Kosagog Tea	5 (0.8%)	Urine therapy	29 (4.7%)
Nutritional Therapy:	68 (11.0%)	Black stone	100 (16.1%)
Mineral treatment	16 (2.6%)	Shark cartilage	15 (2.4%)
Nutri water	43 (6.9%)	Python fat	56 (9.0%)
Honey	347 (56.0%)	Animal extracts	23 (3.7%)
**Spiritual Therapy:**			
Faith/prayer healing	306 (49.4%)	Crude oil	140 (22.6%)
Transcendental meditation	11 (1.8%)		
Visualization	36 (5.8%)		
Psychic Therapy	3 (0.5%)		
Mental imagery	5 (0.8%)		

*Multiple responses given

Major reasons for CAM use as reported by the respondents include their being natural (48.1%) and to promote and maintain health (46.3%). Other reasons (Table 6) include: for quick action (19.0%), conventional medicine has side effects (15.0%), Conventional medicine is expensive (7.7%), and conventional medicine is not effective (7.3%).

**Table 6:** Reasons for using CAM remedies *

S/N	Reasons for Use	Frequency	Percentage
a.	Conventional medicine is not effective	45	7.3%
b.	CAM is quick or fast in action	118	19.0%
c.	Conventional medicine is expensive	48	7.7%
d.	CAM remedies are natural	298	48.1%
e.	Conventional medicine has side effects	93	15.0%
f.	CAM is more in keeping with one's belief and faith	34	7.1.0%
g.	To promote and maintain health	287	46.3%
h.	Others	15	2.4%

*Multiple responses given

## Discussion

Reports on the prevalence of CAM use varies greatly in both developed and developing countries [5,7-9,12]. Figures have ranged from 7% to 83% [5-7], but the average rate across adult studies has been 31.4% [5]. We found a high prevalence (84.7%) of CAM use which is one of the highest reported in literature. This variation could be as a result of what is included under the umbrella of CAM, the nature, cultural values, belief systems, religious underpinnings and practices of our society, as well as the cost and degree of accessibility of conventional medicine. Many Nigerians still utilize traditional medical practices to treat diseases and ailments despite current emphasis on conventional treatment. However, this high prevalence rate does not agree with the study carried out on American adults where a low prevalence of (36%) in 2002, and (38.3%) in 2007 were reported respectively.

Demographic variations associated with prevalence of CAM use have been reported in many studies. Some studies have found associations between age, gender, socioeconomic status, and level of education. Studies in developed countries found that women have higher prevalence of use than men, and that the peak age of use is among young adults/middle aged [4,10]. Also, higher income, higher level of education, and higher socio economic status have been linked to higher prevalence of CAM use. In this study, whereas sex, marital status and level of income were associated with the use of CAM, level of education and age were found to have no relationship with the use of CAM. This finding is supported by findings in other developing countries where age and educational level had no relationship with CAM use [5,7,9].

In this study, males were more inclined to use CAM more than females while usage of CAM increased with low income status. This greater male gender use may be attributed to the African man's strong belief in traditional medicine and his inability to squeeze out time out of their busy schedule to visit the conventional hospitals [7]. In addition, hospitals are perceived by consumers to be time consuming and expensive while CAM is seen as being accessible, affordable and not-time consuming.

Most of the respondents (74.0%) were classified as low income earners and poverty may have contributed to the use of CAM. Nigeria is a developing country with more than 70% of the population living in rural areas and half the population survive on less than 1 dollar per day [21]. In addition, with most CAM products easily accessible and affordable, people are more likely to resort to it than the more expensive conventional therapy coupled with the non-existence of national health insurance scheme. Most of the studies on CAM use in developed countries have been carried out on middle and high income group. This could account for the disparities in the association between socio-demographic and economic profiles and frequency of CAM use.

On the average, five different forms of CAM were used by each adult. This high ratio may reflect an easier access to these CAM products and "fashion" trends over time.

CAM products used by respondents in this study are consistent with most frequently used CAM products in literature [2,5-7]. In addition this study made a remarkable finding in the use of honey as a CAM product which has not been mentioned in the literature of CAM use. The use of honey especially for wound dressing has been reported in United Kingdom and this is attributed to antibiotic-resistant bacteria which have become widespread clinical problem [22]. This has resulted in the availability of the number of honey-based wound treatment/dressings in United Kingdom.

We also found that prayer healing was another common therapy used by the respondents. Religion has always enjoyed high favour with most African communities and this could be responsible for the increased number of people who used prayer/faith healing to ease unfavourable health conditions. Singh, et al, recorded that herbs and spiritual healing were the two most common forms of CAM used among Indians in South Africa [10]. In US, herbal preparations were found to be the most common form of CAM used among the elderly [13].

The use of traditional medicine to treat or relieve symptoms of ill-health has never been far from the lives of an indigenous African population. Combination of many forms of CAM to get all the possible benefits is a common phenomenon. It was not surprising to find many adults in this study use biological products including honey and herbs to improve their quality of life and for treatment of certain diseases. This has given birth to new trends of biomedics under the brand names of forever living products, Aloe Vera, GNLD, Tianshi, etc. This trend which has been there, since the primitive era shows no signs of slowing. The multi-level marketing strategies as well as the advert that portray these products as natural herbs, fruits and food supplement are responsible for their popularity among the black population. The African man's affinity for nature also explains why a significant number of the respondents utilize items like crude oil, black stone, and python fat.

Massage is another form of CAM that was used by respondents. It is fast gaining popularity. As reported by the respondents, it improves muscle tone, circulation and revitalises the body systems.

Major reasons for CAM use as reported by the respondents include their being natural and to promote and maintain health. These findings are in line with other studies that reported the growing public acceptance of herb and other products because they are generally perceived to be more natural with fewer side effect(s), as well as influence well-being and quality of life [5,15]. In contrast, findings from this study did not agree with results of other studies conducted in developing countries [7,11] which reported that most people expect CAM to treat or cure their ailments. This variation could be as a result of the characteristics of the study population. The present study was carried out among adults whose health status was generally satisfactory while studies in other developing countries were conducted among cancer patients who were sick.

## Conclusion

This study has shown a high prevalence of CAM use among the adult populations in Enugu urban. The black man's flare for natural things and the innate urge in man to try new and alternative ways to promote and maintain health supports the high preference for CAM use. The fact that these products are approved by NAFDAC and are easily accessible has increased the perception that they are natural and safe. This trend which is neither static nor decreasing is likely to continue if there is no control by the National drug-safety monitoring system. There is need for regulation by the appropriate authority to ensure evidence of safety, efficacy and rational use of CAM.

Although this study cannot be generalised to the general Nigerian population, it has increased the body of knowledge on CAM use by adult population. Future studies should examine the prevalence of CAM use at the national level and among different socio cultural groups.

## Competing interests

We have no competing financial or non-financial interests in this study except to add to the body of existing knowledge in CAM

## Authors' contributions

JO conceived the study and wrote the first draft of the paper. All the authors participated in the data collection, data analysis and interpretation of data. ILO did the critical revision of the draft. All authors read and approved the final manuscript.

## Pre-publication history

The pre-publication history for this paper can be accessed here:

http://www.biomedcentral.com/1472-6882/11/19/prepub

## Supplementary Material

Additional file 1 **Survey Questionnaire**. Click here for file
