# Supplementary material for: Complementary and alternative medicine use among adults in Enugu, Nigeria
Source: BMC Complement Altern Med. 2011 Mar 4;11:19. doi: 10.1186/1472-6882-11-19 (PMC3066112; doi:10.1186/1472-6882-11-19)
Supplement: Additional file 1 — Survey Questionnaire. [file 1472-6882-11-19-S1.PDF]

Additional file 1: Survey Questionnaire

SECTION A: DEMOGRAPHIC PROFILE

Please tick √ as applicable.

1. Age:

a. 18-25 [ ]
b. 26 – 33 [ ]
c. 34 – 41 [ ]
d. 42 – 49 [ ]
e. 50 – 57 [ ]
f. 58 – 65 [ ]

2. Sex: Male [ ] Female [ ]

3. Marital status: a) Married [ ] b) Not Married [ ]
c) Widow/widower [ ] d) Divorced/separated [ ]

4. Highest level of education attained:
a) No formal education [ ] b) Primary education [ ]
c) Secondary education d) Tertiary education [ ]

5. Estimated level of income per month:
a) Less than N50, 000 per month [ ]
b) N50, 000 – N100, 000 per month [ ]
c) More than N100,000 per month [ ]

6. Religion:
a) Christianity [ ] b) Moslem [ ] c) Traditional religion [ ]

SECTION B

7. How would you rate your overall general health?
a) Satisfactory [ ] b) Good [ ] c) Not good [ ]
d) Seriously sick [ ]

8. Have you used any medicine or remedy other than that given to you by a medical doctor, when you experience any health problems or health needs?
a) Yes [ ] b) No [ ]

9. How many CAM remedies have you used in the past one year?

a) 1 – 5 [ ] b) 6 – 10 [ ] c) 1 – 15 [ ] d) 16 – 20 [ ]

10. Below is a list of CAM people have used for various reasons. We would like to know which ones you have used in the past and which ones you are currently using and the mode of use.

Biological products:

Names of Products	9. Utilization	10. What is the Mode of Use			
	Used and Currently Using	Consume it (orally)	Rob it on body	Say it out/Recite it	Other mode of use of CAM, (Specify)
Herbal drugs					
High dose mega vitamins					
Forever living products					
Tuja 1000					
GNLD Products					
Tianshi products					
Formor Products					
Noni Juice					
Medicinal Tea					
Green Tea					
Kosagog Tea					
Nutritional therapies and supplements					
Mineral treatment					
Nutri water					
Honey					

- Spiritual therapy/mind-body systems

Names of remedies	9. Utilization	10. What is the Mode of Use			
	Used and Currently Using	Consume it (orally)	Rob it on body	Say it out/Recite it	Other mode of use of CAM, (Specify)
Faith/prayer house healing					
Divination/incantation					
Transcendental meditation					
Visualization/vision					
Hypnosis					
Psychic therapy					
Mental imagery					

- Alternative system

Names of remedies	9. Utilization	10. What is the Mode of Use			
	Used and Currently Using	Consume it (orally)	Rob it on body	Say it out/Recite it	Other mode of use of CAM, (Specify)
Chinese Medicine					
Indian Medicine					
Acupuncture					
Homeopathy					
Naturopathy					

- Physical therapy/body manipulations

Names of remedies	9. Utilization	10. What is the Mode of Use			
	Used and Currently Using	Consume it (orally)	Rob it on body	Say it out/Recite it	Other mode of use of CAM, (Specify)
Chiropractic					
Osteopathy/bone setters					
Massage					
Manual healing (therapeutic touch)					

- Energy therapies

Names of remedies	9. Utilization	10. What is the Mode of Use			
	Used and Currently Using	Consume it (orally)	Rob it on body	Say it out/Recite it	Other mode of use of CAM, (Specify)
Bioelectro magnetics					
Oxygen/Ozone treatment					

- Others

Names of Remedies	9. Utilization	10. What is the Mode of Use			
	Used and Currently Using	Consume it (orally)	Rob it on body	Say it out/Recite it	Other mode of use of CAM, (Specify)
Blood letting					
Local Surgery/scarification					
Ritual sacrifice					
Urine therapy					
Folk remedies (Specify)					
Black stone					
Shark cartilage					
Python fat					
Animal extracts					
Crude oil					

11. Are there other types of CAM not listed above which you have used and /or still using?

a) Yes [ ] b) No [ ]

12. If yes, please specify…………………………………………………

13 What are your reasons for using CAM remedies you ticked?

Please tick as many as applies to you

a) Conventional medicine is not always effective [ ]

b) CAM is quick or fast in action [ ]

c) Conventional medicine is too expensive [ ]

d) CAM is natural [ ]

e) Conventional medicine has side effects [ ]

f) CAM is more in keeping with one's belief and faith [ ]

g) To promote and maintain ones health [ ]

j) Others (please specify) …………………………………………
